# Growth hormone is increased in the lungs and enhances experimental lung metastasis of melanoma in DJ-1 KO mice

**DOI:** 10.1186/s12885-016-2898-5

**Published:** 2016-11-08

**Authors:** Chia-Hung Chien, Ming-Jen Lee, Houng-Chi Liou, Horng-Huei Liou, Wen-Mei Fu

**Affiliations:** 1 Institute of Clinical Medicine, National Cheng Kung University, No. 138, Shengli Road, Tainan, 704 Taiwan; 2 Department of Neurology, National Taiwan University Hospital, No. 7, Chung-shan South Road, Taipei, 10016 Taiwan; 3 Pharmacological Institute, College of Medicine, National Taiwan University, No. 1, Sec. 1, Jen-Ai Road, Taipei, 10051 Taiwan; 4 National Institute of Cancer Research, National Health Research Institutes, No. 367, Shengli Road, Tainan, 704 Taiwan; 5 Department of Pharmacology, College of Medicine, National Taiwan University, No. 1, Sec. 1, Jen-Ai Road, Taipei, 10051 Taiwan

**Keywords:** Growth hormone, Melanoma, Lung metastasis, Knockout mice, Oncogenesis

## Abstract

**Background:**

Growth hormone (GH) mainly serves an endocrine function to regulate somatic growth, but also serves an autocrine function in lung growth and pulmonary function. Several recent studies have demonstrated the role of autocrine GH in tumor progression in some organs. However, it is not clear whether excessive secretion of GH in the lungs is related to pulmonary nodule formation.

**Methods:**

Firstly, the lung tissues dissected from mice were used for Western blotting and PCR measurement. Secondly, the cultured cells were used for examining effects of GH on B16F10 murine melanoma cells. Thirdly, male C57BL/6 mice were intravenously injected with B16F10 cells and then subcutaneously injected with recombinant GH twice per week for three weeks. Finally, stably transfected pool of B16F10 cells with knockdown of growth hormone receptor (GHR) was used to be injected into mice.

**Results:**

We found that expression of GH was elevated in the lungs of DJ-1 knockout (KO) mice. We also examined the effects of GH on the growth of cultured melanoma cells. The results showed that GH increased proliferation, colony formation, and invasive capacity of B16F10 cells. In addition, GH also increased the expression of matrix metalloproteinases (MMPs) in B16F10 cells. Administration of GH in vivo enhanced lung nodule formation in C57/B6 mice. Increased lung nodule formation in DJ-1 KO mice following intravenous injection of melanoma cells was inhibited by GHR knockdown in B16F10 cells.

**Conclusions:**

These results indicate that up-regulation of GH in the lungs of DJ-1 KO mice may enhance the malignancy of B16F10 cells and nodule formation in pulmonary metastasis of melanoma.

**Electronic supplementary material:**

The online version of this article (doi:10.1186/s12885-016-2898-5) contains supplementary material, which is available to authorized users.

## Background

DJ-1, a chaperon and anti-oxidative protein plays a crucial role in oncogenesis [1, 2]. In addition, DJ-1 deficiency is related to autosomal recessive Parkinson’s disease [3]. In cancer cells, DJ-1 is known as an oncogene, which reacts with activated Ras [4], a potential serum biomarker secreted from breast cancer cells [1] and malignant melanoma [5]. Overexpression of DJ-1 decreases the expression of Bax and suppresses caspase activation to promote the growth of tumor cells [6]. Moreover, DJ-1 reportedly mediates the phosphatidylinositol 3-kinase (PI3K) survival pathway by negatively modulating the phosphatase and tensin homolog (PTEN) tumor suppressor [7]. In our previous studies, we found that DJ-1 deficiency upregulates levels of IL-1β in the microenvironment of the lungs and enhances metastasis of B16F10 cells in DJ-1 KO mice [8]. Therefore, excess DJ-1 or DJ-1 deficiency in cancer cells or their microenvironment, respectively, can both lead to tumor progression. On the other hand, some studies have indicated that cytokines, such as IL-1β, can promote growth hormone (GH) synthesis and secretion in cultured cells [9, 10]. Thus, we will further examine whether GH also plays a role in lung metastasis of melanoma cells in DJ-1 KO mice.

GH is reported to promote the development of certain cancers. Epidemiological studies indicate that the risk of colorectal cancer is increased in patients with acromegaly and animal studies also demonstrate that up-regulated levels of endogenous GH can cause mammary carcinoma in transgenic mice [11, 12]. GH is mainly secreted from the anterior pituitary and plays an important role in an individual’s development [13]. It binds to the growth hormone receptor (GHR) and exerts its effects through insulin-like growth factor-I (IGF-I) signaling, which controls cell proliferation, survival, and differentiation and enhances cell cycle progression in many cell types [14]. Notably, GH can also be expressed locally in the lungs of rats during fetal and neonatal development [15]. GHRs are expressed in lung epithelia to enable GH effects [16] and IGF-1 is widely expressed during rodent lung organogenesis [17]. These findings indicate that autocrine functions of lung GH may enhance lung growth and survival of surrounding cells. In addition, GH is expressed in human mammary epithelial cells and autocrine GH can promote survival, proliferation, and migration of the human mammary carcinoma cell line MCF-7 and invasive capacity of the human microvascular endothelial cell line (HMEC-1) [18]. However, the autocrine effects of GH on tumor cells in the lungs remain unclear.

GHR (but not GH) is reportedly expressed in melanoma cells; therefore, melanoma cells can respond to GH stimulation [19, 20]. GHR stimulation can promote cell invasion and metastasis [21] and GHR deficiency down-regulates the incidence of cancer [22]. We aimed to examine whether there is a connection between lung GH expression and lung metastasis of GHR-expressing melanoma cells. We found that GH expression was upregulated in the lungs of DJ-1 KO mice, which increased the malignant potential of melanoma cells.

## Methods

### Animals and cell culture

Male C57BL/6 mice as controls were supplied by the Animal Center of Medical College, National Taiwan University. Male DJ-1 KO mice donated by Dr. Tak W. Mak (Toronto, ON, Canada) were on a C57BL/6 background. Mice at 5–6 weeks of age (20–25 g) were used, given free access to food and water, and maintained at an ambient temperature of 25 °C. All animal experiments were reviewed and approved by the Institutional Animal Care and Use Committee of the National Taiwan University. B16F10 murine melanoma cells (from American Type Culture Collection) were maintained in the cell culture in a humidified incubator (5 % CO_2_, 37 °C) in Roswell Park Memorial Institute (RPMI) medium supplemented with 10 % heat-inactivated fetal bovine serum (FBS) (Biological Industries, Kibbutz Beit Haemek, Israel), 100 U/ml penicillin, and 0.1 mg/ml streptomycin (Invitrogen, Carlsbad, CA).

### RNA extraction, semi-quantitative RT-PCR, and real-time quantitative PCR

RNA was extracted from tissues using TRIzol (MDBio Inc., Taipei, Taiwan). Synthesis of cDNA was achieved using MMLV RTase (Promega, Madison, Wisconsin, USA). Synthesized cDNA was used as the template for semi-quantitative RT-PCR and real-time quantitative PCR. The primer sequences were as follows: mouse growth hormone (GH): forward, 5′-CAGCCTGATGTTCGGCACCTCGGA-3′ and reverse, 5′-GCGGCGACACTTCATGACCCGCA-3′; mouse IGF-1: forward, 5′-CTGGACCAGAGACCCTTTGC-3′ and reverse, 5′-AGAGCGGGCTGCTTTTGTAG-3′; mouse GAPDH: forward, 5′-GCCATCAACGCCCCTTCATT-3′ and reverse, 5′-ACGGAAGGCCATGCCAGTGAGCTT-3′. Mouse GH (Mm00433590_g1); IGF-1 (Mm00439560_m1); and GAPDH (Mm99999915_g1) TaqMan probes were purchased from Applied Biosystems (USA). The data were analyzed by the StepOne Real-Time PCR system (ABI, USA). The mRNA levels of GH and IGF-1 were normalized to that of GAPDH and expressed relative to the control using the formula 2^-ΔΔCT^.

### Western blotting

Protein was extracted from tissues using radioimmunoprecipitation (RIPA) buffer containing 150 mM NaCl; 50 mM Tris–HCl; 1 mM ethylene glycol tetraacetic acid (EGTA); 1 % Nonidet P-40; 0.25 % deoxycholate; 1 mM sodium fluoride; 50 mM sodium orthovanadate; 5 mM phenylmethylsulfonyl fluoride (PMSF); 10 μg/ml aprotinin; 10 μg/ml pH 7.5, leupeptin; and Halt protease inhibitor cocktail (Thermo, IL, USA). Protein concentration was determined using the bicinchoninic acid (BCA) protein assay kit (Pierce, Rockford, IL). Bovine serum albumin was used as the standard. Proteins were separated by SDS-PAGE and transferred to polyvinylidene fluoride (PVDF) membranes (Millipore, Billerica, MA). The membranes were soaked in skim milk dissolved in phosphate-buffered saline (PBS) for 1 h to block nonspecific binding and the immune reaction was then allowed to proceed overnight at 4 °C with the following primary antibodies: mouse anti-MMP-2; rabbit anti-MMP-9; rabbit anti-MMP-13 (1:1000; Santa Cruz, CA, USA); rabbit anti-DJ-1 (1:3000; Enzo Life Sciences, UK); goat anti-growth hormone (1:1000; Santa Cruz, CA, USA) goat anti-GHR (1:1000; R&D Systems, Minneapolis, MN, USA); and mouse anti-actin (1:10,000; Chemicon, Temecula, CA). The blots were then incubated with HRP-conjugated secondary antibody (1:10,000; GeneTex, CA, USA). Protein bands were detected using an enhanced chemiluminescence system (Thermo, IL, USA) and quantification was determined by the ImageQuant 5.0 software.

### ELISA analysis of growth hormone

Sera prepared from wild type (WT) and DJ-1 knockout mice were used for the quantitative measurement of GH, using a mouse GH ELISA Kit (Millipore, Billerica, MA, USA) according to the manufacturer’s instructions. Samples were incubated with pre-coated primary GH monoclonal antibody for 2 h. After washing away nonspecifically bound materials, an enzyme-linked polyclonal secondary antibody was added to the wells to form a sandwich complex. A substrate solution was then added to the wells for 30 min to yield color. Finally, a stop solution was added, and the optical density (OD) of each well was measured, using an ELISA reader set at 450 nm. GH concentrations in the samples were then determined by comparing the OD of the samples with the standard curve.

### MTT reaction and BrdU ELISA analysis

B16F10 cells (5 × 10^3^ cells) were seeded on 96-well plates and incubated overnight in RPMI medium supplemented with 10 % FBS. The medium was then replaced with serum-free medium containing GH (0.5, 5, and 15 ng/ml; GenScript, Piscataway, USA). After 24 h incubation, the supernatant was discarded and the MTT solution (0.5 mg/ml, Sigma–Aldrich, St. Louis, MO, USA) was added to each well for thiazolyl blue tetrazolium bromide (MTT) analysis. After 30 min incubation, the MTT solution was discarded and the formazan crystal generated was completely dissolved in dimethyl sulfoxide (DMSO). The absorbance was detected with a spectrophotometer at 570 nm. Following incubation of GH for 24 h, measurement procedures for bromodeoxyuridine (BrdU) ELISA analysis were followed according to manufacturer’s instructions (Roche Applied Science, IN, USA). The incorporation of BrdU was performed for 4 h and chemiluminescent signals produced from the ELISA substrate were measured with a luminescent meter.

### Colony formation

In a six-well culture plate, each well was divided into three layers. The lower layer was 0.7 % solid agarose (3 ml). The middle layer contained B16F10 cells (2 × 10^3^ cells) incubated in 0.7 % solid agarose (1.5 ml); 10 % FBS; RPMI (1.5 ml); and GH (1 and 10 ng/ml). The upper layer was RPMI medium (3 ml) supplemented with 10 % FBS and GH (1 and 10 ng/ml). Twelve days later, the colonies were photographed and counted using an inverted microscope.

### Cell invasion

B16F10 cells (5 × 10^4^ cells) suspended in 10 % FBS RPMI medium were seeded to the cell culture inserts with 8-μm pore polycarbonate filters (Coring, NY). The filters were pre-coated with 25 μL Matrigel (BD Biosciences, Bedford, MA). RPMI medium containing 50 % FBS was used as a chemoattractant in the lower chamber. After 1-h incubation, GH (1 and 10 ng/ml) was then added to upper and lower chambers. Three days later, cells on the upper surface of the filters were removed by wiping with a cotton swab. Cells that penetrated the pores to the lower surface of filters were stained with 0.05 % crystal violet solution (in 20 % methanol). The cells in three random fields per well were photographed and counted using an inverted microscope.

### Pulmonary metastasis

B16F10 cells (6 × 10^4^) were injected into the femoral vein of mice. Three weeks later, the mice were euthanized and lung nodules were photographed and counted using a dissecting microscope.

### Administration of growth hormone and prolactin in mice

Mice were intravenously injected with B16F10 cells (6 × 10^4^). GH or prolactin (5 mg/kg each; R&D system, Minneapolis, MN, USA) were then subcutaneously injected into mice, twice per week for three weeks.

### Murine melanoma cells with knockdown of GHR

B16F10 murine melanoma cells were maintained in RPMI medium supplemented with 10 % heat-inactivated FBS, 100 U/ml penicillin, and 0.1 mg/ml streptomycin (Invitrogen, Carlsbad, CA). Knockdown of GHR in the cells was achieved by transfecting the cells with a plasmid vector carrying shRNA, which targets GHR transcripts (target Sequence: CCCGACTTCTACAATGATGAT), whereas cells transfected with an empty plasmid vector, i.e. pLKO.1, were used as the control. Melanoma cells were transfected with plasmid vectors, using the Oligofectamine reagent (Invitrogen, Carlsbad, CA) dissolved in Opti-MEM medium (Life Technologies, Van Allen Way, Carlsbad, CA, USA). Six hours after transfection, the medium was replaced with RPMI medium, and puromycin (1 ng/ml) was added to the cultured medium to kill cells lacking chromosomal integration of the gene. A stably transfected pool was established following selection with puromycin, and knockdown of GHR was confirmed using Western blot analysis.

### Statistical analysis

Statistical analysis was performed using the Student’s *t*-test. Statistical comparisons of more than two groups were performed using one-way analysis of variance (ANOVA) followed with Bonferroni’s post hoc test. All data were presented as means ± SEM. Differences were considered statistically significant at *P* < 0.05.

## Results

### Increase in growth hormone levels in lung tissue of DJ-1 knockout mice

**Fig. 1:**
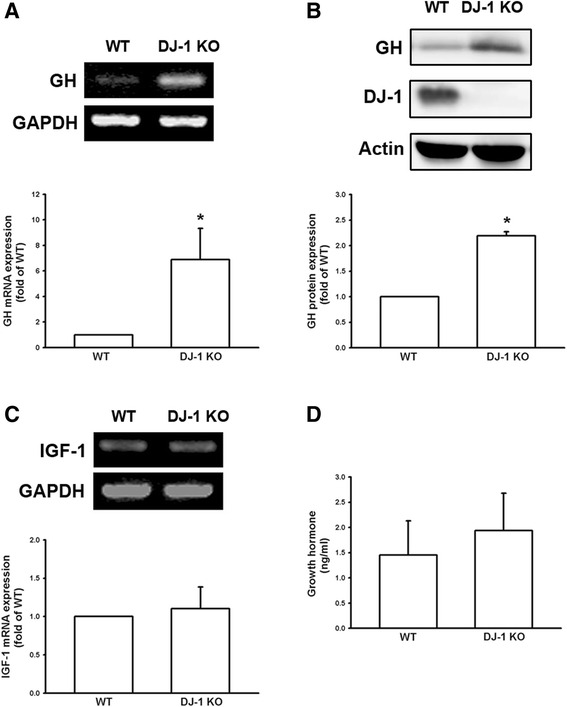
Increase of growth hormone expression in the lungs of DJ-1 KO mice. Lung tissues were isolated from WT and DJ-1 KO mice and used for semi-quantitative PCR (**a**, *upper panel*); real-time quantitative PCR (**a**, *lower panel*); and Western blotting (**b**). Note that there was an increase in GH mRNA and protein expression levels in pulmonary tissue of DJ-1 KO mice. **c** Insulin-like growth factor 1 (IGF-1) mRNA expression in pulmonary tissue and (**d**) serum levels of GH were not significantly different between WT and DJ-1 KO mice. Data are presented as mean ± SEM (*n* = 5 for each group); *, *P* < 0.05 compared to WT. WT: wild type; KO: knockout; GH: growth hormone

We examined mRNA expression of GH in the lungs of DJ-1 KO mice. The results of semi-quantitative PCR (upper panels) and real-time quantitative PCR (lower panels) showed that GH mRNA (Fig. 1a) increased in lung tissue of DJ-1 KO mice and Western blot analysis further confirmed the higher expression of GH protein in the lung tissue of DJ-1 KO mice (Fig. 1b). On the other hand, it has been reported that GH can promote cell survival and proliferation through IGF-1 signaling [14]. We then examined the expression level of IGF-1 in DJ-1 KO mice. The result showed that the mRNA levels of IGF-1 in lungs of mice were not affected in DJ-1 KO mice (Fig. 1c).

Since GH levels were increased in the lung tissue of DJ-1 KO mice, we also measured serum levels of GH. As shown in Fig. 1d, no significant difference was observed in the serum levels of GH between DJ-1 KO and WT mice. These results suggest that GH was elevated locally in the lung tissue of DJ-1 KO mice, but not systemically in the circulation.

### Growth hormone increases cell survival, proliferation, colony formation, and invasive capacity of melanoma cells

**Fig. 2:**
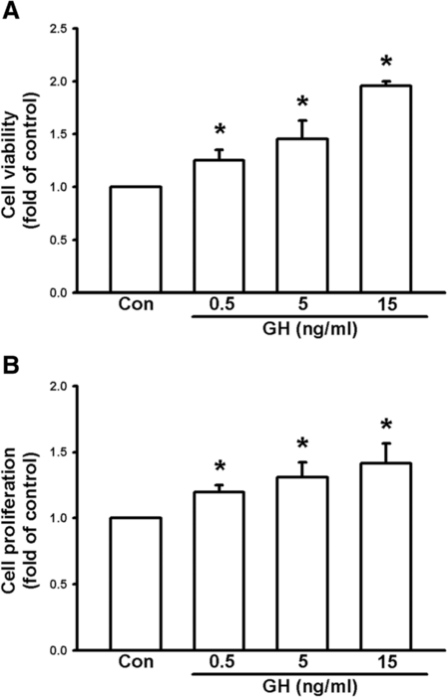
Growth hormone enhances survival and proliferation of B16F10 melanoma cells. **a** Cell viability measured using the MTT assay. Note that treatment of GH (0.5, 5, 15 ng/ml) increased cell viability of B16F10 cells in a concentration-dependent manner. **b** Cell proliferation evaluated using BrdU uptake analysis. Note that treatment of GH (0.5, 5, 15 ng/ml) enhanced cell proliferation in a concentration-dependent manner. Data are presented as mean ± SEM (*n* = 4 for each group); *, *P* < 0.05 compared to the control; BrdU, bromodeoxyuridine

It has been reported that GH has an effect on human cancer cells, such as mammary carcinoma [18]. Moreover, GHRs have been demonstrated in human and murine melanoma cells [20, 23]. We therefore examined the effects of GH on cultured melanoma cells. The MTT assay was used to examine the viability of B16F10 cells, following treatment with recombinant GH protein (at 0.5, 5, and 15 ng/ml) for 24 h. The results showed that GH could enhance the viability of B16F10 cells in a concentration-dependent manner (Fig. 2a). Furthermore, BrdU uptake was used to examine the proliferation of B16F10 cells, following treatment with recombinant GH protein (0.5, 5, and 15 ng/ml) for 24 h. The results showed that GH could also increase the proliferation of B16F10 cells in a concentration-dependent manner (Fig. 2b).

**Fig. 3:**
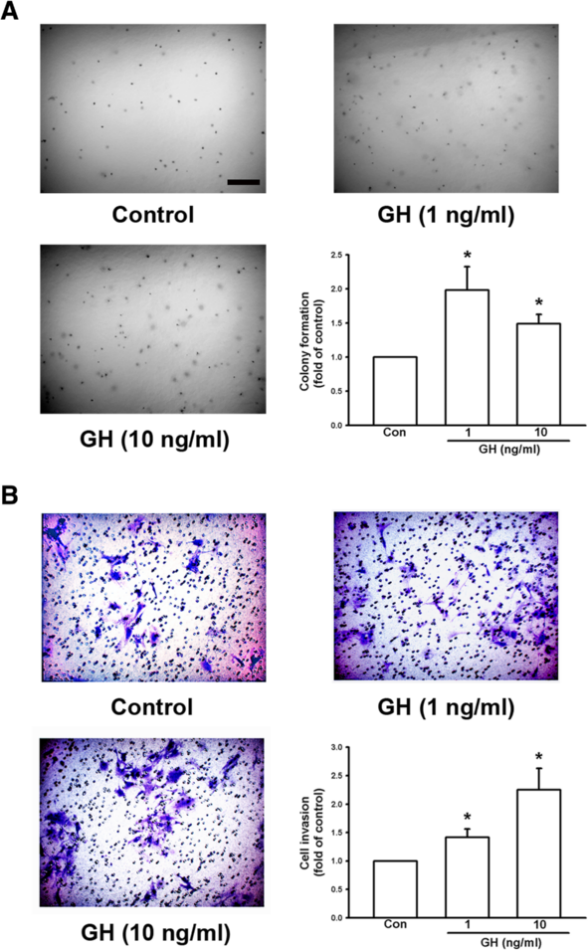
Growth hormone enhances colony formation and invasive capacity of B16F10 melanoma cells. **a** Colony formation of B16F10 cells in soft agar with and without GH. Note that GH increased B16F10 cell colony formation in a concentration-dependent manner and colonies were photographed and counted. **b** B16F10 cells were seeded into a transwell with 8-μm pore polycarbonate filters and matrix gel. Cells penetrated the pores to the lower surface of filters and were stained with crystal violet and counted. The results showed that GH increased the invasive capacity of B16F10 cells in a concentration-dependent manner. Data are presented as mean ± SEM (*n* = 4 for each group); *, *P* < 0.05 compared to the control. Scale bar = 0.2 mm

We further examined the effects of GH on colony formation, which was an in vitro metastasis model. B16F10 cells were seeded in agarose gel and treated with recombinant GH protein for 12 d. The results showed that GH increased colony formation of B16F10 cells (Fig. 3a) up to 1.98-fold at 1 ng/ml GH. In cell invasion analysis, B16F10 cells were seeded on transwell culture inserts with filters, which were pre-coated with Matrigel, and treated with recombinant GH protein. The results showed that treatment with GH for 3 d increased invasion of B16F10 cells in a concentration-dependent manner (Fig. 3b). The invasive capacity of melanoma cells increased up to 2.25-fold at 10 ng/ml GH. These results suggest that GH administration can enhance the malignant potential of B16F10 melanoma cells.

### Growth hormone increases the expression of matrix metalloproteinases in melanoma cells

**Fig. 4:**
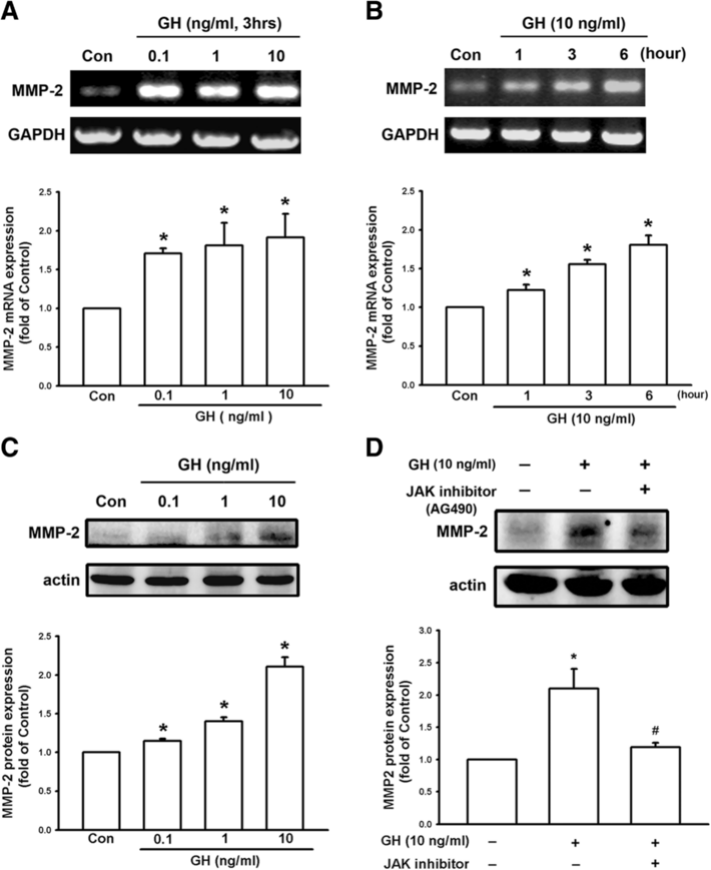
Growth hormone increases the expression of MMP-2 in B16F10 cells through JAK signaling. **a** Expression of MMP2 mRNA was increased in B16F10 cells following 3 h of treatment with GH in a concentration-dependent manner. **b** MMP-2 expression was increased by 10 ng/ml in a time-dependent manner. **c** B16F10 cells treated with GH (0.1, 1, 10 ng/ml) for 6 h increased expression of MMP-2 protein in a concentration-dependent manner. **d** GH-induced increase of MMP-2 was inhibited by JAK inhibitor (AG490). Data are presented as mean ± SEM (*n* = 3 for each group); *, *P* < 0.05 compared to the control (Con); #, *P* < 0.05 compared to GH treatment alone; JAK, Janus kinase

**Fig. 5:**
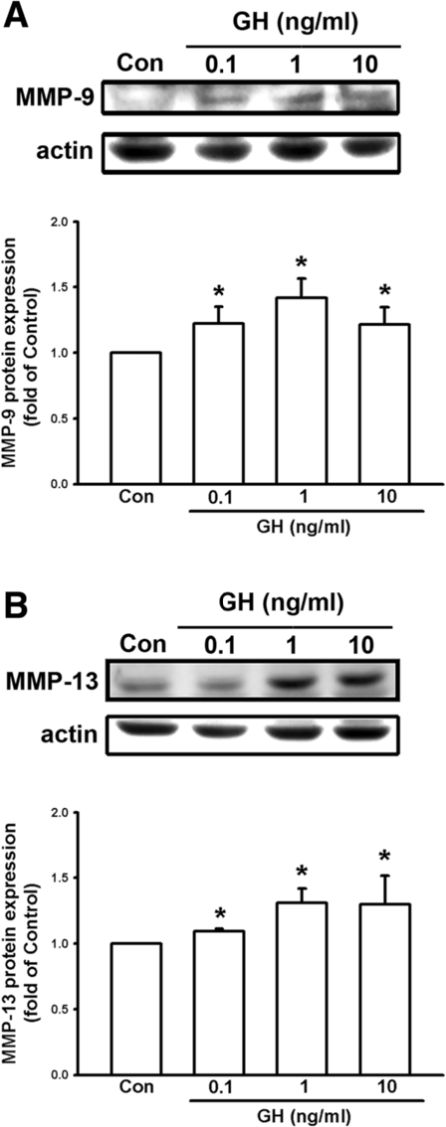
Growth hormone enhances the expression of MMP-9 and MMP-13 in B16F10 cells. The protein levels of MMP-9 (**a**) and MMP-13 (**b**) in B16F10 cells were up-regulated in a concentration-dependent manner following GH treatment (0.1, 1, 10 ng/ml) for 6 h. Data are presented as mean ± SEM (*n* = 3 for each group); *, *P* < 0.05 compared to the control (Con)

Some members of the MMP family play a role in tumor cell invasion because their effects can lead to degradation of the extracellular matrix. We therefore examined whether MMP levels were enhanced by treatment with GH. B16F10 melanoma cells were treated with various doses (0.1, 1, and 10 ng/ml) of GH for 3 h (Fig. 4a), and then at various time intervals (0, 1, 3 and 6 h) at GH 10 ng/ml (Fig. 4b; F = 22.362, *P* < 0.05). Cells were collected and mRNA expression of MMP-2 was examined using RT-PCR. The results showed that GH increased the expression levels of MMP-2 mRNA in a concentration- and time-dependent manner. We then treated B16F10 cells with GH (0.1, 1, and 10 ng/ml) for 6 h and proteins were prepared for Western blotting. It was found that GH also increased expression of MMP-2 protein (Fig. 4c; F = 27.471, *P* < 0.05) in a concentration-dependent manner. According to previous reports, GH binds to GHRs and activates nonreceptor tyrosine kinase, Janus kinase 2 (JAK2), resulting in cellular effects [24]. We then used the JAK2 inhibitor, AG490 (Santa Cruz, CA, USA) to examine whether it can antagonize the effect of GH. The results showed that the GH-induced MMP-2 expression was down-regulated by the treatment of JAK2 inhibitor (Fig. 4d). In addition, we found that GH also enhanced the protein expression of MMP-9 (Fig. 5a) and MMP-13 (Fig. 5b) in a concentration-dependent manner. These results suggest that GH may enhance the invasive capacity of B16F10 cells, by upregulating the expression of matrix metalloproteinases.

### Growth hormone increases lung nodule formation in C57/B6 mice

**Fig. 6:**
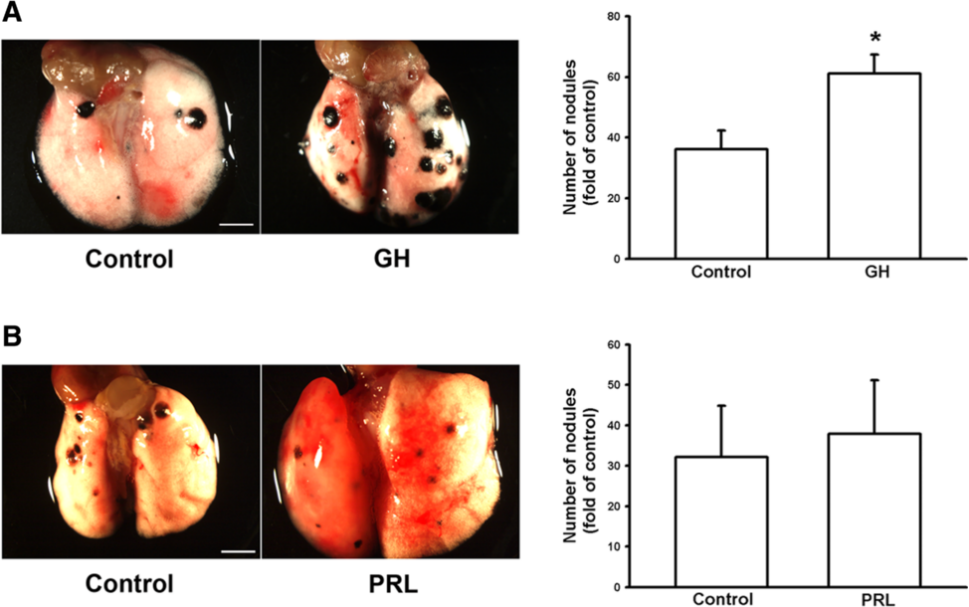
Growth hormone enhances lung nodule formation in C57/B6 mice. B16F10 cells (6 × 10^4^ cells) were injected into the femoral vein of C57/B6 mice. The mice were subcutaneously injected with GH (**a**) or prolactin (**b**) (5 mg/kg, twice/week, respectively). The lung nodules were photographed and counted three weeks later. Results showed an increase in the number of lung nodules in C57/B6 mice following GH administration and there was on difference after prolactin administration. Data are presented as mean ± SEM (*n* = 11–12 for each group in **a**; *n* = 5 for each group in **b**); *, *P* < 0.05 compared to the control. Scale bar = 3 mm

Since GH can enhance the viability, proliferation, colony formation, and invasive capacity of melanoma cells in vitro, B16F10 cells (6 × 10^4^) were injected into the femoral vein of C57/B6 mice, which were then subcutaneously injected with GH (5 mg/kg, twice/week). Mice were sacrificed and lung tissues were isolated three weeks later. We found that treatment with GH increased the number of lung nodules by 1.69-fold (Fig. 6a). Since prolactin is also secreted by the anterior pituitary hormone and serves autocrine functions [25] and prolactin receptor is expressed in melanoma cells [19], we also examined the effect of prolactin on melanoma growth in vivo. The results showed that subcutaneous injection of prolactin (5 mg/kg, twice/week) had no significant effect on lung nodule formation following intravenous injection of melanoma cells in WT mice (Fig. 6b). These results suggest that GH but not prolactin can enhance lung nodule formation of intravenous melanoma cells.

### Increased lung nodule formation in DJ-1 KO mice is inhibited by knockdown of GHR in melanoma cells

**Fig. 7:**
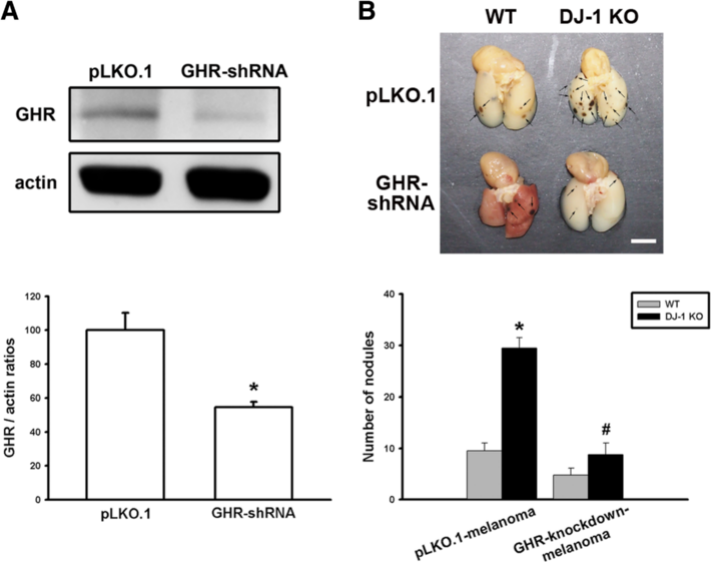
Elevated lung nodule formation in DJ-1 KO mice is suppressed following injection of GHR-knockdown melanoma cells. **a** Western blots showed the knockdown of GHR in B16F10 melanoma cells. *Upper panel*: representative blots of GHR and actin in cells stably transfected with empty plasmids (pLKO.1) or plasmids encoding GHR shRNA. *Lower panel*: bar chart showing statistical results of the Western blot. **b** B16F10 cells (6 × 10^4^) transfected with pLKO.1 plasmids (pLKO.1-melanoma) or GHR shRNA plasmids (GHR-knockdown-melanoma) were intravenously injected into mice. Three weeks later, mice were sacrificed. Gross images (*upper*) showed the melanoma nodules (*arrows* in the panel) and bar chart (*lower*) showed the summarized results of lung nodule numbers in WT and DJ-1 KO mice. Note that melanoma nodule formation was enhanced in DJ-1 KO mice following injection of pLKO.1-melanoma cells, but was suppressed following injection of GHR-knockdown melanoma cells. Data are presented as mean ± SEM (*n* = 5 for each group); *, *P* < 0.05 compared to the control, WT mice with pLKO.1-melanoma; #, *P* < 0.05 compared to DJ-1 KO mice with pLKO.1-melanoma. Scale bars = 0.5 mm

Since GHR is expressed in melanoma cells [19] and GH can enhance the malignant effects of B16F10 melanoma cells in vitro and lung metastasis in vivo, we then used GHR-knockdown B16F10 cells to examine the effects of lung GH in DJ-1 KO mice. As demonstrated by Western blot analysis, expression of the GHR protein was significantly reduced in the cell pool that was stably transfected with shRNA-plasmids (GHR-shRNA) in comparison to the pool of empty plasmids (pLKO.1). This finding suggests that stable knockdown of GHR was successfully established in B16F10 melanoma cells (Fig. 7a). Following intravenous injection of GHR-knockdown B16F10 cells to both WT and DJ-1 KO mice, the increased lung nodule formation in DJ-1 KO mice was inhibited (Fig. 7b). These results suggest that up-regulation of GH in DJ-1-deficient lungs plays a role in promoting the formation of lung nodules.

## Discussion

In the present study, we demonstrated that mRNA and protein levels of GH were increased in the lungs of DJ-1 KO mice (Fig. 1). Furthermore, GH can increase the viability, proliferation, and colony formation of melanoma cells (Figs. 2 and 3). We also found that GH could up-regulate the expression of matrix metalloproteinases, which promote the invasive capacity of melanoma cells (Figs. 3, 4 and 5). Furthermore, we found that treatment with GH increases lung nodule formation, following intravenous injection of melanoma cells in wild-type mice (Fig. 6) and increased lung nodule formation in DJ-1 KO mice can be inhibited by intravenous injection of GHR-deficient melanoma cells (Fig. 7).

B16F10 melanoma cells were used because they are poorly immunogenic and do not express GH [19, 26], so that we can rule out any GH-derived effects caused by cancer cells. Moreover, several studies have shown that melanoma cell lines express high levels of growth hormone receptor and respond to GH treatment. On the other hand, DJ-1 KO mice were used because melanoma or breast cancer is increased in patients with Parkinson's disease according to accumulating epidemiological data [27]. We here thus further explored the connection between cancer and the neurodegenerative disease. Notably, Tillman et al. reported that DJ-1 could directly regulate the activity of the androgen receptor to promote the progression of prostate cancer [28]. Flutamide, an androgen receptor antagonist, can increase the expression of DJ-1 in prostate cancer cell lines by increasing DJ-1 protein stabilization [29]. Another study also indicated that blocking an androgen receptor with flutamide enhances secretion of GH [30]. These results demonstrate that DJ-1 can mediate the progression of hormone-regulated cancer and suggest that there may be a connection between DJ-1 and GH. In the present study, we found that with DJ-1 deficiency, there was a concurrent increase in GH in lung tissue. The relationship among GH, DJ-1, and androgen receptor inhibition requires further investigation.

According to previous studies, GH has a half-life in the serum of only 4–20 mins in animals and human and basal serum level in mice is 8.7 ± 6.5 (<20 ng/ml) [31, 32]. As shown in Fig. 1d, we found that the mean serum level of GH (0.77–2.68 ng/ml) was in the range as reported previously, so that the serum level of GH was normal in DJ-1 knockout mice. In human, the basal level of hGH was 0.63 ± 0.91 [33]. However, unlike in murine lung, GH does not play a direct physiological role in growth and maturation of human lung [34]. Therefore, there may be some species differences in the regulation of lung development by GH. Here we found that expression level of GH was increased in lungs of DJ-1 KO mice as compared with those of WT mice (Fig. 1). Moreover, we also found that levels of GH were up-regulated in spleen and liver of DJ-1 KO mice (Additional file 1: Figure S1). According to former reporters, spleen can produce GH to enhance the maturation of myeloid progenitor cells and liver is a major target organ of GH [35, 36]. However, further experiments are needed to examine their roles and effects in DJ-1 KO mice. As shown in Figs. 2, 3, 4 and 5, we found that GH not only promoted cell proliferation (Fig. 2) but also enhanced cell invasion (Fig. 3). Matrix metalloproteinases upregulation may be involved in the increase of invasion ability (Figs. 4 and 5). Therefore, the increase of lung nodules is not simply a product of increased proliferation induced by GH. GH can enhance other malignant effects of B16F10 cells when tumor cells initiate and grow.

As reported previously, the most common experimental metastasis model is vein injection, which primarily results in lung metastases [37]. Furthermore, it was found that B16-F10, chosen in our study, formed only lung tumor nodules after intravenous injection, whereas B16-F1 yielded some extrapulmonary tumor nodules [38]. Therefore, it demonstrates that lung is the main metastatic organ for B16F10 cells. Despite the increased levels of GH in spleen and liver of DJ-1 KO mice, we focus on the lung nodules formation following intravenous injection of B16F10 cells (Figs. 6 and 7). Notably, a stably transfected pool in cell culture was established following puromycin selection. According to previous studies, a puromycin-resistant gene is commonly used as a selection marker in mammalian cells, since puromycin is toxic to the growth of mammalian cells [39]. Therefore, we infer that puromycin treatment might slightly affect malignant effects of B16F10 melanoma cells, so that the lung nodules were reduced in results of Fig. 7 as compared with the injection of non-treated B16F10 cells. Moreover, the use of pLKO.1-melanoma cells as control cells can rule out any effects caused by puromycin selection procedures. However, the possible cause of less nodule formation needs further examination. In addition, Sustarsic et al. (2013) has reported that melanoma express the highest level of GHR among several human cancer cells of NCI60 panel (US National Cancer Institute’s Development Therapeutics Program). Other studies have also shown that targeting GHR can control cancer metastasis, such as pancreatic cancer [40]. In this study, we found that GHR down-regulation reduced lung metastasis of melanoma cells (Fig. 7). However, it needs further examination to verify whether up-regulated expression of GHR in melanoma can increase the incidence of lung metastasis. Furthermore, we found that the source of increased GH was autocrine from lung tissue of DJ-1 KO mice and the cancer cells were affected by the paracrine GH. According to previous reports, GH can increase STAT5 phosphorylation in metastatic melanoma cells and STAT5 activation is associated with enhanced invasion and metastasis of melanoma [19]. Therefore, we infer that the GH-overexpressing cancer cells should enhance tumor metastasis in control mice as similar as the injection of normal cancer cells in DJ-1 KO mice.

In fact, we found that GH treatment did not affect body weight of mice (data not shown). According to the former studies, GH has been shown to enhance immune function by starting both neutrophils and macrophages for production of cytokines and superoxide anions [41, 42]. GH also acts as a cytokine that induces survival and proliferation of lymphoid cells through the PI-3 kinase/Akt pathway and NF-κB [43]. Moreover, exogenous GH increases levels of superoxide in alveolar macrophages [44]; increases production of NF-κB [45] and lung phosphorylase A activity [46]; suppresses glutathione peroxidase (GPX) and manganese superoxide dismutase (MnSOD) protein levels and activity [47]; and stimulates tyrosine phosphorylation of specific proteins in lung epithelial cells [16]. These effects of GH can all make cancer cells more invasive in the microenvironment of the lung, by reducing antioxidative defense and enhancing inflammatory signaling. However, it was found that GH can not increase the influx of neutrophils into lungs by measuring MPO activity and not enhance lung microvascular permeability by using Evan’s blue dye [45]. Taken together, we infer that there is an altered immune-microenvironment in lungs by GH treatment, which enhances lung metastasis of B16F10 cells through up-regulating levels of superoxide in alveolar macrophages and inhibiting expression of superoxide dismutase enzyme. In addition, our results showed that there was no significant difference in serum levels of GH between WT and DJ-1 KO mice, regardless of whether they had been injected with B16F10 cells (Additional file 1: Figure S2). Therefore, we can infer that GH might play a local inflammatory role that promotes the growth of tumor cells, particularly in the early stages of lung metastasis.

A link between inflammation and cancer has been observed in tumor biopsy specimens [48]. The mechanisms underlying the connection between inflammation and tumorigenesis have been studied within the last decade. The balance between immunosurveillance and tumor-promoting inflammation is quite important in the tumor microenvironment [49]. Inflammatory cells producing reactive oxygen species (ROS), cytokines, chemokines, MMPs, and prostaglandin E2 (PGE2) amplify the signaling cascade of inflammation [50]. Furthermore, some reports suggest that mitochondrial ROS is an important intermediate in inflammation-associated cancer [51]. DJ-1 mutant-associated Parkinson’s disease is in fact related to mitochondrial dysfunction and up-regulation of ROS [52]. Using gelatin zymography, we also found that DJ-1 deficiency enhanced the activity of MMP-2, MMP-9, and MMP-13 in lung tissue (Additional file 1: Figure S3). Therefore, inflammatory reactions and factors promoting the development of cancer cells may exist in DJ-1 KO mice, which might explain why increased metastasis to the lungs can be readily observed in DJ-1 KO mice. Nevertheless, further research is necessary to determine whether up-regulated activity of matrix metalloproteinases is correlated with GH levels in the lungs of DJ-1 KO mice.

## Conclusion

In summary, both mRNA and protein levels of GH are increased in the lungs of DJ-1 KO mice. Moreover, GH can enhance the malignant effects of B16F10 cells and play a regulatory role in nodule formation of melanoma in lung metastasis. Furthermore increased lung nodule formation in DJ-1 KO mice is inhibited by intravenous injection of GHR-deficient melanoma cells.

## Additional file

Additional file 1:
**Figure S1**. Up-regulated expression of growth hormone in spleen and liver of DJ-1 KO mice. **Figure S2**. Serum levels of growth hormone do not be affected in mice injected with B16F10 cells. **Figure S3**. Enhanced enzyme activity of MMP proteins in lungs of DJ-1 KO mice. (DOC 691 kb)

## Abbreviations

GH: Growth hormone
GHR: Growth hormone receptor
IGF-I: Insulin-like growth factor-I
KO: Knockout
MMPs: Matrix metalloproteinases
RT-PCR: Reverse transcription polymerase chain reaction
shRNA: Short hairpin ribonucleic acid
WT: Wild type
