# Supplementary material for: Growth hormone is increased in the lungs and enhances experimental lung metastasis of melanoma in DJ-1 KO mice
Source: BMC Cancer. 2016 Nov 8;16:871. doi: 10.1186/s12885-016-2898-5 (PMC5101681; doi:10.1186/s12885-016-2898-5)
Supplement: Additional file 1: — Figure S1. Up-regulated expression of growth hormone in spleen and liver of DJ-1 KO mice. Figure S2. Serum levels of growth hormone do not be affected in mice injected with B16F10 cells. Figure S3. Enhanced enzyme activity of MMP proteins in lungs of DJ-1 KO mice. (DOC 691 kb) [file 12885_2016_2898_MOESM1_ESM.doc]

**Supportive information**


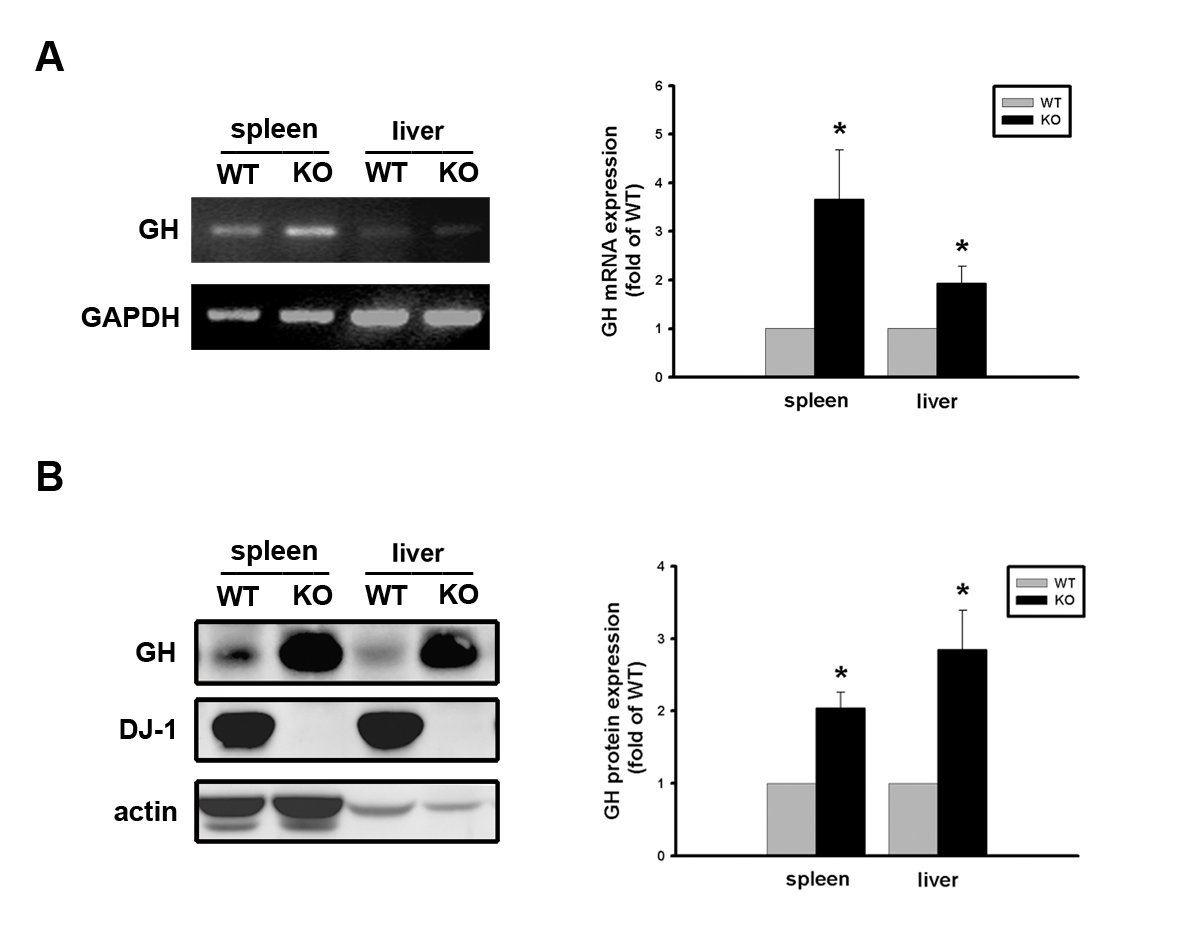


**Supp. Fig. 1. Up-regulated expression of growth hormone in spleen and liver of DJ-1 KO mice**

Spleen and liver tissues were isolated from WT and DJ-1 KO mice and used for semi-quantitative PCR (A, left panel); real-time quantitative PCR (A, right panel); and western blotting (B). Note that there was an increase in expression levels of GH mRNA and protein in spleen and liver tissue of DJ-1 KO mice. Data are presented as mean ± SEM (n = 5 for each group); *, P < 0.05 compared to WT. WT: wild type; KO: knockout; GH: growth hormone


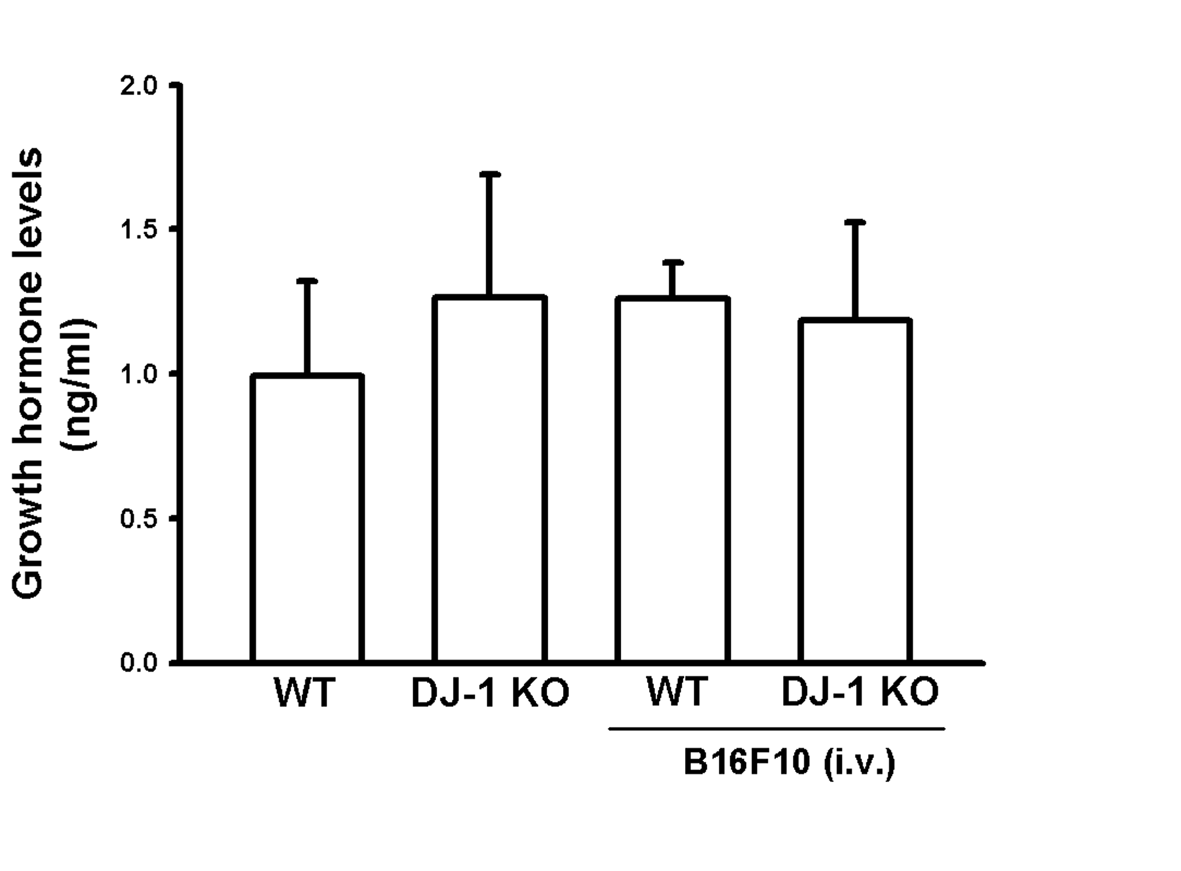


**Supp. Fig. 2. Serum levels of growth hormone do not be affected in mice injected with B16F10 cells**

There was no significant difference in the serum levels of growth hormone between WT and DJ-1 KO mice, regardless of whether they had been injected with B16F10 cells. Data are presented as mean ± S.E.M. (n = 5 for each group). i.v. = intravenous injection


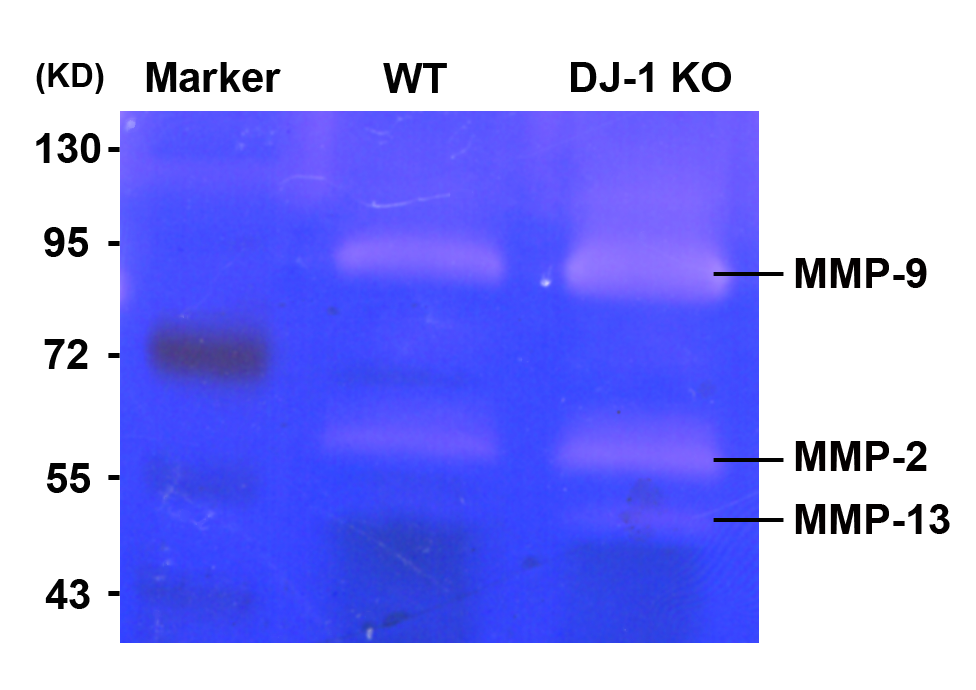


**Supp. Fig. 3. Enhanced enzyme activity of MMP proteins in lungs of DJ-1 KO mice**

The lungs were isolated from untreated WT and DJ-1 KO mice for performing gelatin zymography. The bands of MMP-2, MMP-9 and MMP-13 are indicated. Note that knockout of DJ-1 increased the more enzyme activity of MMP-2 (1.69-fold), MMP-9 (1.82-fold) and MMP-13 (4.76-fold) in lungs as compared with those in WT mice.
